# A Lipid Based Antigen Delivery System Efficiently Facilitates MHC Class-I Antigen Presentation in Dendritic Cells to Stimulate CD8^+^ T Cells

**DOI:** 10.1038/srep27206

**Published:** 2016-06-02

**Authors:** Mithun Maji, Saumyabrata Mazumder, Souparno Bhattacharya, Somsubhra Thakur Choudhury, Abdus Sabur, Md. Shadab, Pradyot Bhattacharya, Nahid Ali

**Affiliations:** 1 Indian Institute of Chemical Biology, Infectious Diseases and Immunology Division, 4, Raja S.C. Mullick Road, Jadavpur, Kolkata-700032, India

## Abstract

The most effective strategy for protection against intracellular infections such as *Leishmania* is vaccination with live parasites. Use of recombinant proteins avoids the risks associated with live vaccines. However, due to low immunogenicity, they fail to trigger T cell responses particularly of CD8^+^ cells requisite for persistent immunity. Previously we showed the importance of protein entrapment in cationic liposomes and MPL as adjuvant for elicitation of CD4^+^ and CD8^+^ T cell responses for long-term protection. In this study we investigated the role of cationic liposomes on maturation and antigen presentation capacity of dendritic cells (DCs). We observed that cationic liposomes were taken up very efficiently by DCs and transported to different cellular sites. DCs activated with liposomal rgp63 led to efficient presentation of antigen to specific CD4^+^ and CD8^+^ T cells. Furthermore, lymphoid CD8^+^ T cells from liposomal rgp63 immunized mice demonstrated better proliferative ability when co-cultured *ex vivo* with stimulated DCs. Addition of MPL to vaccine enhanced the antigen presentation by DCs and induced more efficient antigen specific CD8^+^ T cell responses when compared to free and liposomal antigen. These liposomal formulations presented to CD8^+^ T cells through TAP-dependent MHC-I pathway offer new possibilities for a safe subunit vaccine.

There is an enormous need to develop technologies that are safe and capable of delivering antigens efficiently to antigen presenting cells (APCs) to promote a wide range of cellular immune responses. The strategies to deal with these issues may have a significant impact on the prevention of microbial, pathogenic and viral infections including many life-threatening diseases^1^,^2^,^3^. Several vaccine formulations in development are aimed to induce strong CD8^+^ along with effector CD4^+^ T cell responses for effective clearance of intracellular pathogens^4^,^5^. To this end live vaccine vectors have been developed which have the capacity to stimulate strong immune responses. However, various safety issues with live vectors complicate further development of this technology^6^,^7^. The focus on vaccine designing has therefore now been shifted towards the development of non-living synthetic vaccines composed of one or a few selected protein antigens^8^,^9^. However, most of these protein-based antigens are less immunogenic and are rapidly degraded by proteases resulting in poor presentation by MHC molecules^10^. They, therefore, fail to stimulate T cell responses required for protection against pathogens such as HIV, malaria, tuberculosis and *Leishmania*^11^. To improve antigen persistence, uptake and presentation, along with supplementing immune stimulation, co-administration of safe and efficient adjuvants is essential. The combination of delivery systems and immunopotentiating adjuvants has therefore emerged as a promising strategy for rationale vaccine design^12^,^13^. A number of techniques such as micelles, liposomes, archaeosomes, polymersomes and ISCOMs have been used to deliver protein antigens to professional APCs^14^,^15^,^16^,^17^,^18^,^19^. Cationic liposomes are particularly more attractive and promising as delivery vehicles owing to their low immunogenicity, safety in clinical use, depot effect and simplicity of preparation^20^,^21^,^22^. Moreover, these liposomes are very efficiently taken up by APCs^23^. While particulate delivery vehicles like cationic liposomes ensure the delivery of entrapped antigens to APCs, immunopotentiators including different TLR agonists stimulate immune cells through particular receptors^24^. MPL (Monophosphoryl lipid A), a TLR agonist and an immunopotentiating agent already in use in some approved vaccines, has been widely studied^5^,^12^,^25^. Previously, we have shown that immunization of a model animal with liposome encapsulated leishmanial antigens alone or with adjuvant induced protective T cell responses^26^. Although our earlier studies demonstrated promise for this delivery technology, until now the uptake and intracellular routing of liposomes by APCs to stimulate CD4^+^ and CD8^+^ T cell responses have not been shown.

Distearoyl phosphatidylcholine (DSPC), due to its high transition point, along with helper lipid cholesterol can be formed into stable liposomes that avoid easy clearance from blood. Addition of positively charged moieties improves both entrapment as well as retaining efficiency of biological macromolecules along with enhancing uptake by APCs^12^,^23^,^27^. This allows persistence of antigens for durable uptake and presentation needed for long term immunity for diseases like leishmaniasis^28^,^29^. In this study, we report that DSPC bearing cationic liposomes were efficiently taken up by bone-marrow derived dendritic cells (BMDCs), and transported to different intracellular compartments. Liposomal entrapment of leishmanial recombinant gp63 (lrgp) induced antigen presentation by DCs for specific T cell recognition and subsequent proliferation. Interestingly, mixing of MPL-trehalose dicorynomycolate (MPL-TDM) with lrgp significantly enhanced the CD8^+^ T cell response compared to lrgp alone both *in vitro* and *in vivo*. On a mechanistic level, decrease in CD8^+^ T cell proliferation in TAP1 silenced DCs suggests that liposomal antigens are presented through a TAP-dependent MHC-I pathway. These results, therefore, demonstrate the potentiality of this formulation as a promising antigen delivery technology for stimulation of T cell responses.

## Results

### Immunostimulation of BMDCs by DSPC bearing cationic liposomes

**Figure 1:**
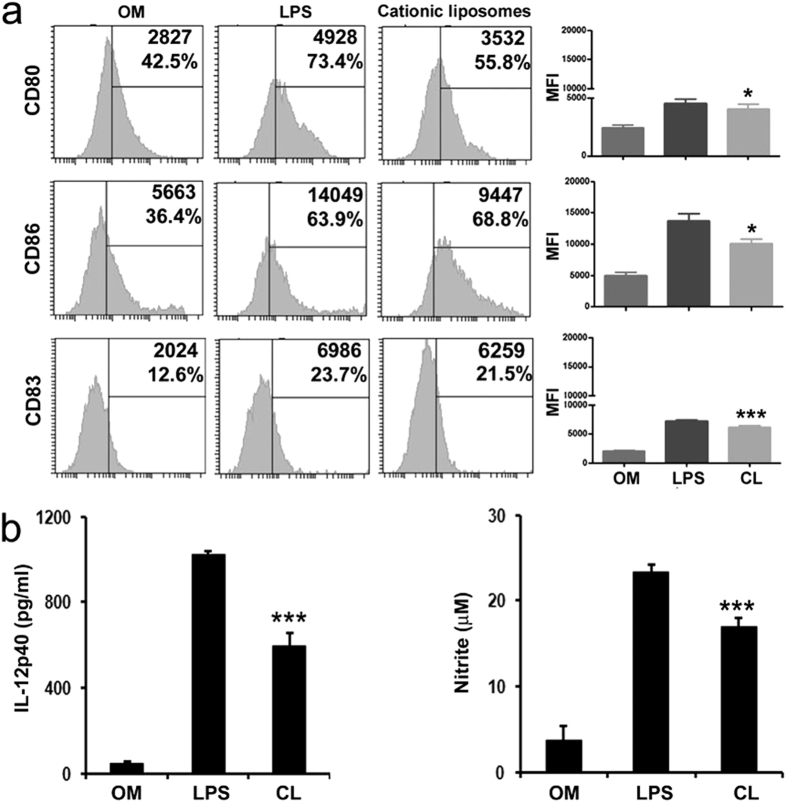
Immunostimulation and maturation of BMDCs with cationic liposomes. Cultured bone-marrow derived DCs (day 6) were stimulated with only media, LPS (1μg/ml) and cationic (CL) liposomes (100 μM liposome concentration) for 24 h. **(a)** Afterwards cells were immunostained with antibodies against CD11c as a dendritic cell marker and assessed for expression of the co-stimulatory molecules CD80, CD86 and CD83 by flow cytometry. For the analysis, CD11c+ cells were gated first before gating on the selected markers. Numbers inside the histogram represent MFI values and percent positive cells. Corresponding graph in right panel summarizes the MFI values of three experiments. *p < 0.05, ***p < 0.001 analyzed by one-way ANOVA, followed by Tukey’s multiple comparison test, compared to untreated control. **(b)** Stimulated dendritic cell culture supernatants were removed at 48 h for IL-12*p*40 quantification and nitric oxide (NO) production. Data represent triplicate mean ± SE. ***p < 0.001 analyzed by one-way ANOVA, followed by Tukey’s multiple comparison test, compared to untreated control.

Expression of surface co-stimulatory molecules and the production of cytokines are the important characteristics of maturation of DCs^30^,^31^. To examine whether the liposomes could stimulate the phenotypic maturation of DCs *in vitro*, DCs were incubated with characterized (Table 1) DSPC bearing cationic liposomes (100 μM) or LPS (as positive control) for 24 h and the levels of surface expression of co-stimulatory molecules (CD80, CD86 and CD83) were determined by flow cytometry. Compared to untreated DCs, stimulated DCs became positive for CD83 population (DC maturation marker), and also showed upregulated expression of CD80 and CD86. As shown in Fig. 1a, analysis of mean fluorescent Intensity (MFI) values reveals that expression of CD80, CD86 and CD83 were significantly upregulated in cationic liposome treated DCs compared to only media control (*p* < 0.05). To determine whether the upregulation of co-stimulatory molecules could stimulate DCs to produce cytokines (another indication of DC maturation), DCs were incubated with liposomes or LPS for 48 h, followed by actual measurement of IL-12p40 and nitric oxide (NO) levels in culture supernatants. As shown in Fig. 1b, DCs stimulated with cationic liposomes significantly upregulated the production of IL-12p40 and NO compared to unstimulated control (*p* < 0.001). Thus, on the basis of the capacity to upregulate the expression of surface co-stimulatory molecules, as well as to promote the IL-12p40 and NO production in DCs, it can be concluded that positively charged liposomes activate and promote the maturation of DCs efficiently.

### Analysis of uptake and intracellular trafficking of fluorescently labeled cationic liposomes

In view of our observations on the activation and maturation of DCs by cationic liposomes (Fig. 1), we next examined the uptake and intracellular localization of fluorescent labeled liposomes using fluorometric methods. DCs were stimulated with cationic liposomes. Further to compare the intracellular localization we used differentially charged fluorescent liposomes. Table 2 shows the results after 18 h stimulation of DCs with HPTS labeled cationic liposomes, anionic liposomes and neutral liposomes (HPTS-CL, -AL, -NL). DCs took up a considerable amount of HPTS-CL and more efficiently (higher fluorescence intensity) than other liposomes (Table 2 and Supplementary Figure S1a,b and Table S1). Additionally, determination of the fluorescence ratio of 450 nm/405 nm indicates that cationic liposomes were delivered to cellular sites with a neutral or mildly acidic pH, whereas anionic and neutral liposomes were processed to cellular compartments with acidic pH inside DCs.

**Figure 2:**
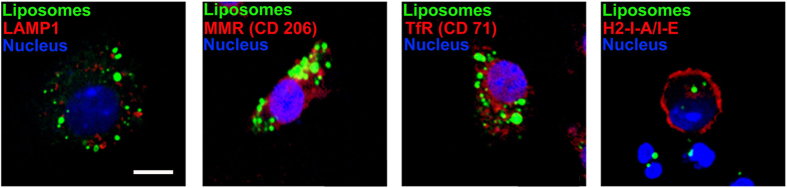
Intracellular trafficking of cationic liposomes in BMDCs. Mouse BMDCs were incubated with rhodamine123 labeled cationic liposomes for 20 h. After incubation, unbound liposomes were washed out, and cells were mounted on slides and stained intracellularly (for details, see “Materials and Methods”) with antibodies against LAMP-1, CD206, CD71 and H2-I-A/I-E. Results are shown as rhodamine123 labeled cationic liposomes (excitation for green), antibody staining (excitation for red), and DAPI staining for nucleus of the cell (excitation for blue). Yellow color indicates localization of liposomes with specific compartments. (Scale bar = 10 μm).

Confocal laser scanning microscopy (CLSM) was performed to study the localization of cationic liposomes within DCs. After 20 h incubation with rhodamine123 labeled cationic liposomes on slides, DCs were washed to remove excess fluorescent liposomes and stained with antibodies against different sub-cellular markers (lysosome associated membrane protein-1 (CD107a/LAMP-1) to visualize late endosomal and early lysosomal compartments, macrophage mannose receptor (MMR/CD206) to visualize phagosomal and early endosomal compartments, transferrin receptor (TfR/CD71) to visualize endosomal compartments and H2-I-A/I-E to visualize major histocompatibility complex-II compartments). Analysis of confocal images revealed that very few cationic liposomes were localized with endosomal compartments (mainly in CD206) and majority of the liposomes did not co-localize with any of the labeled cellular compartments studied (Fig. 2 and Supplementary Figure S2). Thus, only a few of the vesicles gained access to the early endosomal compartments while a majority were either in free cytosol or in some unidentified neutral compartment of DCs as indicated in Table 2. Therefore, it can be envisaged that cationic liposomes may deliver entrapped antigens for efficient presentation by both MHC II and MHC I in DCs.

### *In vitro* stimulation of T cells by cationic liposome encapsulated antigen

**Figure 3:**
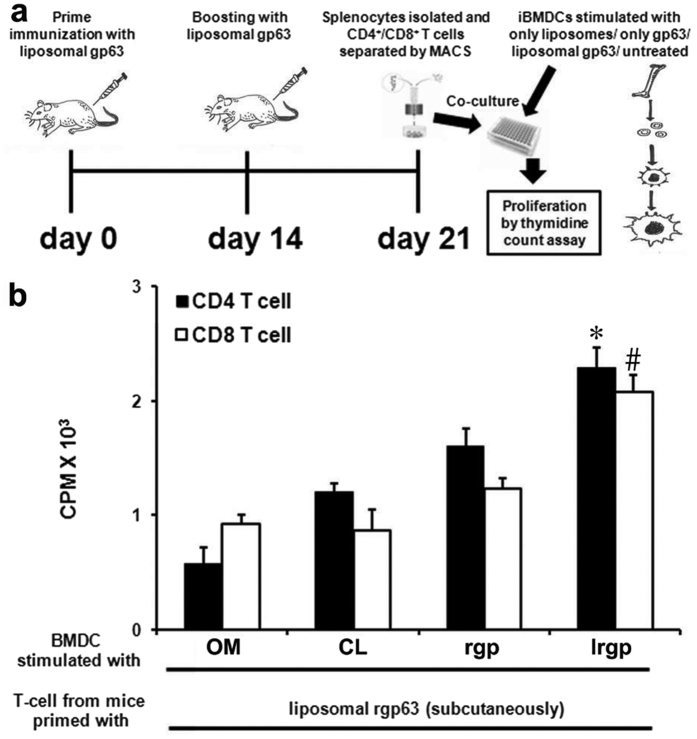
*In vitro* proliferation of CD4^+^ and CD8^+^ T cells in response to presentation of cationic liposome encapsulated leishmanial recombinant gp63 (lrgp63). (**a**) Schematic representation of the experimental setup. (**b**) BMDCs were pulsed with only rgp63 (rgp) or with liposome encapsulated rgp63 (lrgp) for 18 h. DCs cultured in media alone (OM) or pulsed with empty cationic liposomes (CL) were treated as controls. After incubation DCs were washed and co-cultured with splenic CD4^+^ and CD8^+^ T cells, isolated from mice that were immunized earlier with lrgp (for details, see Materials and Methods). T-cell proliferation was monitored by measuring the uptake of thymidine. Data represent triplicate mean CPM × 10^3^ ± SE. *p < 0.05 compared to CD4 T cell response of all the other groups and ^#^*p* < 0.05 compared to CD8^+^ T cell response of all the other groups, assessed by one-way ANOVA, followed by Tukey’s multiple comparison test.

Efficient uptake and internalization of cationic liposomes by DCs reveals that this formulation may serve as a potential vehicle for delivery of vaccine candidates to APCs. However, to generate strong T cell-mediated immune response encapsulated proteins need to be released from the liposomes, processed and trafficked for presentation with MHC molecules for successful stimulation and proliferation of both CD4^+^ and CD8^+^ T cells^17^,^32^. Therefore, we investigated whether entrapment of antigen in liposomes could activate the lysosomal pathway of antigen processing in DCs to present the processed antigens to CD4^+^ T cells. Additionally, the occurrence of cationic liposomes in the cytoplasm of DCs (Table 2 and Fig. 2) indicates that encapsulation of antigen in these liposomes might favor the stimulation of CD8^+^ T cells. To investigate this, we examined T cell proliferation using T cells from mice (that were previously immunized with lrgp subcutaneously), and BMDCs pulsed differentially (see details in “Material and Methods”). Interestingly, DCs were able to present both lrgp and rgp to CD4^+^ T cells, but the capacity to process and present lrgp were significantly higher (*p* < 0.05) than the free protein (Fig. 3). Similarly, as expected from earlier experiments (Table 2 and Fig. 2; delivery of liposomes to cytosol) lrgp pulsed DCs induced proliferation of CD8^+^ T cell higher than DCs pulsed with an equivalent amount of only protein (*p* < 0.05). Additionally, incubation of DCs with only liposomes or media did not show any significant T cell proliferation (Fig. 3). Therefore, these results indicate that encapsulation of protein antigens to cationic liposomes favors the activation of MHC-II pathway of DCs to present processed antigen to CD4^+^ T cells. More interestingly, it also activates MHC-I pathway to present processed antigen to CD8^+^ T cells.

### Stimulation of CD8^+^ T-cells by cationic liposome encapsulated antigens *in vivo*

**Figure 4:**
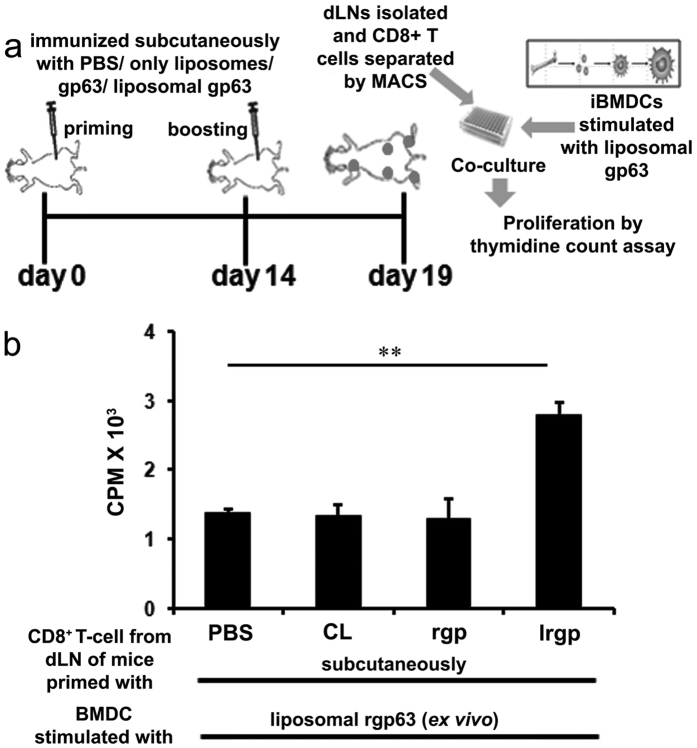
Induction of CD8^+^ T cells *in vivo* by cationic liposome encapsulated antigen. **(a)** Schematic outline of the experimental protocol. **(b)** Groups of five mice were injected subcutaneously with PBS, empty cationic liposomes (CL), 2.5 μg rgp63 (rgp) alone or encapsulated in cationic liposomes (lrgp). Animals were boosted on day 14 with the same antigen preparations received initially and 5 days later draining lymph nodes (dLN) were removed. CD8^+^ T cells from dLN suspensions were purified magnetically using MACS. 1 × 10^5^ CD8^+^ T cells were incubated with 1 × 10^4^ BMDCs (10:1 ratio) that had been incubated with liposomal rgp63 (2.5 μg/ml) for 18 h. T cell proliferation was measured on day 4. Data represent triplicate mean CPM × 10^3^ ± SE. **p < 0.01 analyzed by one-way ANOVA followed by Tukey’s multiple comparison tests.

To examine the correlation of this *in vitro* immunogenicity of cationic liposome entrapped protein with *in vivo* stimulation, we compared the capacity of rgp63 alone, entrapped in cationic liposomes and empty liposomes to induce CD8^+^ T cell responses in naïve mice. In this experiment, BALB/c mice were immunized two times subcutaneously with the above mentioned formulations carrying an equal amount of rgp63 as was administered within the liposomal rgp63. On day 5 after booster, CD8^+^ T cells were prepared from draining lymph nodes (dLN) and then co-cultured with liposomal rgp63 stimulated DCs, which had demonstrated significantly higher CD8^+^ T-cell proliferation *in vitro* (Fig. 3). Interestingly, mice immunized with lrgp induced significantly higher CD8^+^ T cell proliferation compared to equivalent amount of only rgp63 and as well as to control mice immunized with PBS or cationic liposomes alone (Fig. 4). Therefore, these results suggest that cationic liposomes can efficiently deliver encapsulated protein antigen to the APCs for the stimulation of CD8^+^ T-cell responses *in vivo* through cross-presentation.

### Positively charged liposomes carrying antigens and mixed with MPL-TDM elicits potentially stronger CD8^+^ T-cell responses

**Figure 5:**
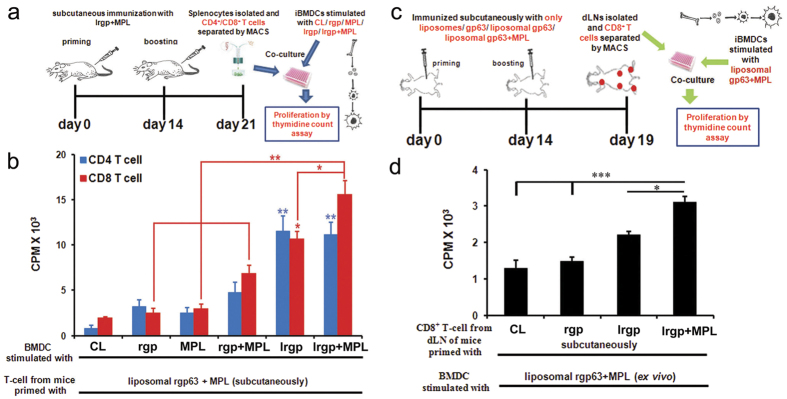
Cationic liposome encapsulated antigen when mixed with TLR agonist superiorly elicits stronger CD8^+^ T-cell response. (**a,c**) Representation of the *in vitro* and *in vivo* experimental setups, respectively. (**b**) Enriched BMDCs were stimulated with 2.5 μg of rgp63 free, entrapped in liposomes or liposomal rgp63 mixed with MPL-TDM. BMDCs stimulated with empty cationic liposomes (CL; 100 μM) or with only MPL-TDM (MPL; 100 ng/ml) or with only rgp63 + only MPL-TDM (rgp + MPL-TDM) served as controls. After 18 h 1 × 10^4^ BMDCs were co-cultured with 1 × 10^5^ CD4^+^ T/CD8^+^ T cells isolated from mouse immunized with lrgp + MPL-TDM (for detail see “Materials and Methods” section). After 4 days of culture, T- cell proliferation was measured by thymidine uptake. (**d**) BALB/c mice were injected subcutaneously on day 0 and 14 with cationic liposomes (CL; 100 μM), 2.5 μg free rgp63 (rgp), liposomal rgp63 alone (lrgp) or mixed with MPL-TDM (lrgp + MPL). On day 5, after the final immunization draining lymph nodes were removed. The LN cell suspensions were prepared, and the CD8^+^ T cells were purified magnetically using MACS. 1 × 10^5^ CD8^+^ T cells were incubated with 1 × 10^4^ BMDCs (10:1 ratio) that had been incubated with lrgp + MPL for 18 h. T cell proliferation was measured on day 4. Data represent mean CPM × 10^3^ ± SE of triplicate cultures. *p < 0.05, **p < 0.01 ***p < 0.001, analyzed by one-way ANOVA followed by Tukey’s multiple comparison tests.

Recently, toll-like receptor (TLR) agonists were used as vaccine adjuvants in association with liposomal formulations to augment vaccine efficacy especially to generate sustained cellular immunity^26^,^33^. Thus, to generate enhanced immunological responses using liposome encapsulated antigens we mixed MPL-TDM with the formulation. MPL, an agonist for TLR-4 expressed on APCs and other cells, and capable of amplifying immunological responses, is the first Food and Drug Administration approved immunostimulant used in licensed vaccines^34^,^35^. MPL-TDM when mixed with cationic liposomes induced the upregulation of surface co-stimulatory molecules (CD40, CD86) and production of IL-12p40 and nitric oxide (Supplementary Figure S3) on DCs *in vitro*, compared with only media or MPL-TDM stimulated DCs. In addition, when we checked out the proliferative responses *in vitro* (Fig. 5a, for detail see “Materials and Method” section), we found that DCs incubated with lrgp or liposomal rgp63 mixed with MPL-TDM (lrgp + MPL) induced not only CD4^+^ T cell proliferation but also triggered CD8^+^ T cell proliferation, as measured by thymidine incorporation (^3^H-TdR) assay. However, although both these formulations induced comparable level of CD4^+^ T cell proliferation, DCs stimulated with lrgp + MPL remarkably triggered significant CD8^+^ T cell proliferation, compared not only to DCs pulsed with MPL-TDM (*p* < 0.001), free gp63 (*p* < 0.001) or rgp63 + MPL-TDM (*p* < 0.01) but also with lrgp (*p* < 0.05) (Fig. 5b). Conversely, very poor CD8^+^ T cell responses were detected when DCs were stimulated with only gp63, MPL-TDM alone or empty liposomes, indicating the specificity of CD8^+^ T cell responses stimulated by lrgp + MPL. To test the functionality of T cells, we assessed the ability of *ex vivo* stimulated CD8^+^ T cells to produce Th1cytokines. CD8^+^ T cells from mice immunized with lrgp + MPL when co-cultured with lrgp + MPL stimulated DCs had significantly higher levels of IFN-γ and IL-2 competent CD8^+^ T cells than even lrgp stimulated DCs (Supplementary Figure S4). These results indicate that mixing of MPL-TDM with liposomes not only triggers the maturation of DCs but also induces proliferation and production of Th1 cytokines from CD8^+^ T cell, as predicted with TLR adjuvanted particulate vaccine delivery systems.

To investigate the influence of MPL-TDM mixed liposomal formulation on CD8^+^ T cell responses *in vivo*, we immunized BALB/c mice subcutaneously with empty cationic liposomes, free rgp63, lrgp alone or mixed with MPL-TDM on day 0 and boosted on day 14 with the same formulations. Five days after the final immunization CD8^+^ T cells were isolated from dLNs and co-cultured with BMDCs that were previously pulsed with lrgp + MPL (Fig. 5c), the formulation which triggered robust CD8^+^ T cell proliferation *in vitro* (Fig. 5b). Mice immunized with lrgp + MPL induced significantly stronger CD8^+^ T cell responses (validates *in vitro* findings) compared to empty liposomes (*p* < 0.001), only gp63 (*p*<0.001) and more importantly lrgp (*p* < 0.05) (Fig. 5d). Thus, upregulated T cell responses with liposome encapsulated antigens mixed with TLR-4 ligand MPL-TDM, compared to other formulations, could be attributed to improving the delivery of antigens to APCs, stimulation of DCs and enhanced antigen presentation through MHC complexes.

### Differential expression of molecules associated with MHC-I and MHC-II pathways of antigen processing

**Figure 6:**
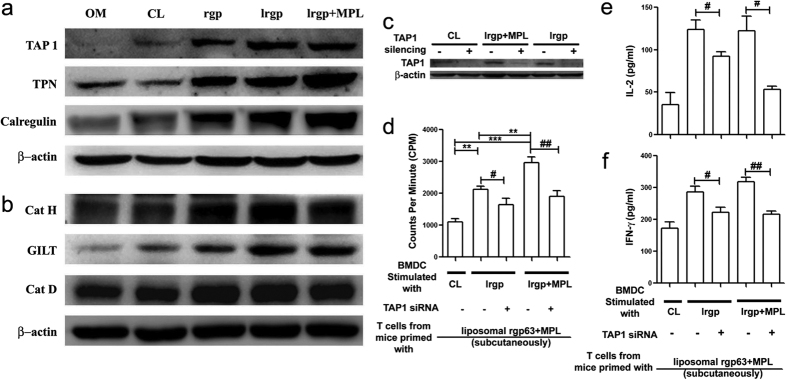
BMDCs differentially express MHC class-I and II pathway associated molecules of antigen processing machinery and knocking down of TAP1 in DCs abrogates presentation of liposomal protein to CD8^+^ T cell. (**a,b**) BMDCs were stimulated with only gp63, liposomal gp63 and liposomal gp63+MPL. Unstimulated DCs (OM) and DCs stimulated with only liposome (CL) served as controls. (**a**) Western blots for Tap-1, Tapasin and Calregulin on extracts of stimulated BMDCs. (**b**) Western blots for Cathepsin H, Gilt and Cathepsin D on extracts of stimulated BMDCs. β-actin was used as loading control. (**c–f**) Mouse BMDCs were transfected with either control or TAP1 siRNA followed by stimulation with liposomal protein alone or with MPL-TDM or cationic liposomes (served as experimental controls). (**c**) Expression of TAP1 abrogation in siRNA treated BMDCs was evaluated by Western blot analysis. After 18 h of stimulation cells were co-cultured with isolated CD8^+^ T cells from mice that were immunized earlier with liposomal gp63+MPL-TDM. (Details in “Materials and Methods” section). (**d**) T cell proliferation was assayed by thymidine uptake as described earlier and mean CPM of triplicate cultures from a representative experiment are shown. *p < 0.05, **p < 0.01, ***p < 0.001 assessed by one-way ANOVA, followed by Tukey’s multiple comparison test. (**e**) IL-2 and (**f**) IFN-γ measured from collected culture supernatants. Results are representative of three individual experiments and the error bars represent mean ± SE (n = 3). ^#^*p* < 0.05, ^##^*p* < 0.01 by Student’s *t*-test.

To investigate the antigen processing machinery involved in the presentation of exogenous liposomal antigens to CD4^+^ and CD8^+^ T cells, we stimulated the BMDCs with cationic liposomes, rgp63 alone, lrgp or lrgp + MPL and checked the expression of different components that regulate MHC class I and MHC class II- restricted antigen presentation pathways in DCs. Analysis of real-time PCR (Supplementary Figure S5, Table S2) and western blotting data revealed that the differentially stimulated DCs expressed components of the MHC-I and MHC-II processing pathways, consistent with their capacity to stimulate CD4^+^ and CD8^+^ T cell responses. MHC-I and MHC-II processing associated proteins were expressed at higher levels in DCs stimulated with lrgp alone or mixed with MPL-TDM compared to only media, liposomes and only protein. More interestingly, expression of MHC class I pathway associated proteins (TAP-1, Tapasin, Calregulin) were expressed more strongly in lrgp + MPL stimulated DCs compared to lrgp alone, whereas MHC-II pathway associated proteins (Cathepsin H, GILT, Cathespin D) were expressed to comparable levels in both lrgp alone and with MPL-TDM (Fig. 6a,b and Supplementary Figure S6). Therefore, these results suggest that the expressions of proteins involved in processing and presentation of antigens (lrgp or lrgp + MPL) by BMDCs were consistent with their ability to stimulate CD4^+^ and CD8^+^ T cell responses (Figs 3).

### Cationic liposome encapsulated antigens presented to CD8^+^ T cells by a TAP-dependent pathway

Earlier Van Kaer *et al*.^36^ demonstrated that TAP1 (−/−) mice were deficient in antigen presentation through MHC class I. Thus, to confirm that cationic liposome encapsulated protein was presented on MHC class I molecules through a TAP-dependent pathway to stimulate CD8^+^ T cells, normal and TAP1 silenced (Fig. 6c and Supplementary Figure S7) BMDCs were pulsed with only cationic liposomes, lrgp alone or with MPL-TDM and co-cultured with CD8^+^ T cells from mice that were immunized earlier with lrgp + MPL. Figure 6d–f demonstrate that TAP1 silenced DCs stimulate proliferation of CD8^+^ T cells less efficiently than wild-type DCs, proving the significance of the presence of TAP 1 molecules for delivering cationic liposome encapsulated antigens to the MHC class I pathway for the activation of CD8^+^ T cells.

## Discussion

Development of an efficient delivery system that specifically targets the professional APCs for processing and presentation of encapsulated antigens through MHC complexes to induce broad spectrum T cell responses is a prerequisite to provide resistance against global infections like tuberculosis, malaria, leishmaniasis, viral diseases as well as cancers^11^,^37^,^38^,^39^. Antigens entrapped in DSPC bearing cationic liposomes represent a unique strategy for protection and delivery of encapsulated antigens to DCs for the stimulation of antigen-specific cellular immune responses. In our earlier studies, we reported that antigen encapsulated in liposomes can induce protective immune responses against experimental visceral leishmaniasis, but the pathways of immunostimulation were not known^15^,^26^. Cationic liposomes as opposed to negative and neutral liposomes exhibit a predominant depot effect at the injection site which helps to deliver trapped antigens to APCs more strongly, and this appears to be very much important to induce protective Th1 type of immunological responses^15^,^21^. But, the underlying mechanisms behind this induced T cell response is not fully understood^23^. Herein we have shown that DSPC bearing cationic liposomes were taken up very efficiently by BMDCs and induced their maturation by upregulating the expression of co-stimulatory molecules with enhanced IL-12p40 and NO production. This is important because maturation of DCs usually results in upregulation of T-cell stimulatory capacity^40^. Further the fluorometric studies suggest that besides endosomal localization cationic liposomes can be targeted to intracellular compartments with neutral pH, most likely the cytosol of DCs. This is particularly significant because cytosolic delivery is a critical step for MHC class I antigen presentation for the sequential elevation of antigen-specific CD8^+^ T cell responses and is essential for protection against infectious diseases like leishmaniasis and others^23^,^41^.

Leishmaniasis is a disease complex caused by several species of the members of the protozoan parasite genus *Leishmania*. Visceral form of leishmaniasis is fatal if not treated, and development of a long-term immunity based vaccine remains a major challenge. gp63, a major surface glycoprotein of *Leishmania* spp., has been found to be an important vaccine candidate against visceral leishmaniasis^42^. Recently, a bio-informatics study suggests that a leishmanial gp63 based protein vaccine can be used as a candidate antigen for all forms of leishmaniasis, and is also found to be an important protein member of Leishvacin®^43^,^44^. Of note, entrapment of model antigens has been extensively investigated for the development of vaccines, and tested using model antigen-specific T cell lines^10^,^45^,^46^. Nonetheless, Steinman had urged that “dominant use of OVA as a model antigen may distort the standard for discovering safe, defined, protein-based vaccines”^47^. Taking all these in consideration, we have used leishmanial rgp63 as a model antigen for successful encapsulation into cationic liposomes and studied the antigen-specific T cell responses both *in vitro* and *in vivo* in an animal model. Interestingly, our findings demonstrate that cationic liposome entrapped antigen was intracellularly processed and presented by BMDCs to stimulate antigen-specific CD8^+^ and CD4^+^ T cells, resulting in superior T cell proliferative responses compared to only protein stimulation.

In recent times, several adjuvants have been combined with vaccine candidates to enhance the efficacy of poorly immunogenic antigens and also to induce synergistic or additive T cell responses not sufficiently effective in absence of adjuvants or both with subunit vaccines^15^,^33^,^48^. MPL, a less toxic derivative of LPS and a safe alternative, is currently utilized in clinical trials due to the generation of a wide range of T cell responses when used as an adjuvant^48^. Here, we also found that association of MPL-TDM with liposomal proteins induced CD4^+^ T cell response which is comparable to liposomal gp63 without MPL-TDM. Interestingly, both *in vitro* and *in vivo* studies demonstrated that MPL-TDM adjuvanted liposomal gp63 elicited stronger CD8^+^ T cell responses which were significantly higher than the responses of liposomal protein alone. In contrast, weak T cell responses were observed when stimulated with gp63 or MPL-TDM alone. These findings indicate the specificity of the adjuvanted liposomal formulation to enhance MHC-I presentation of the entrapped antigen. Additionally, we found that a very small amount of liposome-encapsulated antigen can induce higher T cell responses when compared to the equal amount of free protein, *in vitro* and *in vivo*. Therefore, a critical advantage of this delivery technology is that small amounts of antigens will be required for immunization which can effectively stimulate CD4^+^ as well as CD8^+^ T cell responses. These results indicate that inclusion of adjuvants with the recombinant antigens can substantially induce the T cell responses to lower the amounts of proteins, a consequence of an obvious manufacturing benefit^48^.

In the development of new adjuvanted vaccine formulations the information on mechanism of action plays an essential role to understand the possible advantage that these formulations offer^48^. From *in vitro* experiments, antigen encapsulated liposomal formulations were found to trigger the expression of MHC class I associated molecules in DCs (Fig. 6a). Additionally, lack of antigen presentation by TAP1 silenced BMDCs confirmed that cationic liposome encapsulated antigens with MPL-TDM gain access to the MHC class I pathway through the TAP 1 transporter (Fig. 6c–f). This ability of cationic liposomes alone or with adjuvant to target exogenous antigens to the cytosolic MHC class-I pathway is much like the recent evidence of TAP-dependent cross-presentation pathway identified for the presentation of exogenous antigen^38^,^49^,^50^. Elegant, *in vitro* experiments with cultured DCs suggest that antigen presentation to MHC class-II is regulated through the control of various proteases^51^,^52^. Liposomal protein alone or mixed with MPL-TDM, in addition to MHC class-I associated molecules, upregulated the expression of MHC class-II associated proteases (Fig. 6b). These results suggest that liposomal antigen with essential presentation through MHC class-II pathway has the capacity to potentially cross-present the entrapped antigens through a TAP-dependent MHC class-I pathway in DCs, a mechanism which is enhanced in association with MPL-TDM. These results provide preliminary information on the mechanism of action of MPL-TDM adjuvanted DSPC bearing cationic liposomal formulation which may help to understand the potential usefulness that this adjuvant offers for T cell vaccination strategy. Our findings, therefore, provide new possibilities for the development of an immunization tool aimed against diseases such as viral, microbial as well as cancers that also require potentiation of CD4^+^ and, more importantly, CD8^+^ T cell responses.

## Materials and Methods

### Reagents and Mice

Distearoyl phosphatidylcholine (DSPC), cholesterol, phosphatidic acid (PA), lipopolysaccharide (LPS), monophosphoryl-lipid A + trehalose dicorynomycolate adjuvant (MPL-TDM), bovine serum albumin (BSA) and rhodamine 123 were obtained from Sigma (St. Louis, MO). Stearylamine (SA) was obtained from Fluka (Fluka, Buchs SG). 8-Hydroxypyrene-1,3,6-trisulfonic acid trisodium salt (HPTS) and *p*-xylene-bis-pyridinium bromide (DPX) were obtained from Molecular Probes (Eugene, OR). Recombinant mouse granulocyte-macrophage colony stimulating factors (rmGM-CSF) and interleukin-4 (mrIL-4) were obtained from R&D Systems, Inc. (Minneapolis, MN). Tritiated thymidine (^3^H-TdR, specific radioactivity 5 Ci/mmol) was purchased from Amersham Biosciences. Antibodies used for flow cytometric studies were purchased from BD/Pharmingen (San Diego, CA) including PE-Cy7-conjugated anti-mouse CD11c, FITC-conjugated anti-mouse CD86, APC-conjugated anti-mouse CD80 and PE-conjugated anti-mouse CD40. FITC-conjugated anti-mouse CD83 and APC-conjugated anti-mouse MHC-II antibodies were purchased from eBioscience (San Diego, CA). All the antibodies used for confocal immunofluorescence microscopy were purchased from Santa Cruz Biotechnology, Inc. (Dallas, TX).

Four to six weeks old BALB/c mice were obtained from Council of Scientific and Industrial Research-Indian Institute of Chemical Biology (CSIR-IICB, Kolkata, India) animal house. All mice were maintained in the institute facilities under pathogen-free conditions with water and food given *ad libitum*. All the protocols for the animal studies were done in accordance with the guidelines of the Committee for the Purpose of Control and Supervision on Experimental Animals (CPCSEA), Ministry of Environment and Forest, Govt. of India, and approved by the Animal Ethics Committee (147/1999/CPSCEA) of CSIR-IICB.

### Preparation of liposomes and entrapment of gp63 in liposomes

Cationic liposomes were prepared using DSPC, cholesterol and SA at a molar ratio of 7:2:2. Composition of neutral and anionic liposomes were DSPC and cholesterol (7:2 molar ratio), and PA (7:2:2 molar ratio), respectively. Lipid mixtures were dissolved in methanol-chloroform solution and evaporated to dryness in a round bottom glass flask to make a thin film of lipid. Empty and antigen entrapped liposomes were prepared by dispersion of lipid film in either 1ml of PBS alone or containing 500 μg/ml of rgp63 (cloned and purified as described previously^53^; briefly, full-length gp63 cloned into pET16b vector, and for expression *E. coli* BL21 (DE3) pLysS was transformed with pET16bLdgp63).The mixture was then vortexed and sonicated for 30 s in an ultrasound probe sonicator (Misonix; New York, USA) twice with a 1 min gap in between, followed by incubation at 4 °C for 2 h. The excess of free recombinant gp63 was removed by centrifugation at 100,000 *g* for 1h at 4 °C. The amount of protein entrapped in liposomes was estimated by Lowry’s method using BSA as standard in the presence of 0.8% SDS and with appropriate blanks. Entrapment of a pH sensitive, water soluble fluorescent probe, HPTS along with DPX, was performed as described earlier^54^. For localization studies, fluorescent liposomes were prepared with the above mentioned liposomal constituents along with 0.1 mg/ml of rhodamine123 as a lipophilic marker. The lipid film was dispersed in PBS and excess dye was removed from labeled liposomes by centrifugation.

### Characterization and protein entrapment efficiency of liposomes

DSPC bearing lipid vesicles were stored at 4 °C and characterized by undertaking the analysis of vesicle size and zeta potential. The particle size and zeta potential of the liposomes were determined at room temperature by dynamic light scattering using a Malvern Zetasizer Nano-ZS instrument (Malvern, U.K.) by diluting the formulations in doubly filtered (0.22 μm pore size) distilled water. The quoted sizes are the z-average means of the liposomal hydrodynamic diameters (nm). To measure antigen entrapment efficiency, supernatants obtained after centrifugation were collected and protein content estimated by the method described by Lowry *et al*.^55^. When subtracted from initial amount of antigen used for encapsulation, efficiency of rgp63 entrapment was calculated (Table 1).

### BMDC culture

BMDCs were generated on a previously reported method with some modifications^56^,^57^. Briefly, bone marrow cells were cultured in complete RPMI 1640 medium supplemented with 15 ng/ml mouse recombinant GM-CSF and 10 ng/ml mouse recombinant IL-4. On day 4, non-adherent cells were removed, and adherent cells were cultured in fresh medium containing GM-CSF and IL-4. Subsequently on day 6 or 7, immature DCs were incubated in fresh medium with different liposomal formulations, LPS or MPL-TDM at specific concentrations for 18–72 h at 37 °C in a humidified CO_2_ (5%) incubator before analysis of IL-12p40 and nitric oxide (NO) production, expression of surface co-stimulatory markers, and their capacity of antigen presentation.

### Cytokine and NO quantitation

Culture supernatants of differentially stimulated BMDCs were collected and stored at −70 °C for analysis of cytokine and NO production. Measurement of IL-12p40 levels was carried out as detailed in the instructions provided with cytokine ELISA kit (BD Biosciences). The accumulation of NO in the stimulated culture supernatants were measured as described previously^58^. Briefly, 50 μl of the supernatant was mixed with an equal volume of Griess reagent (1% sulfanilamide and 0.1% N-1-naphthylethylene diamine hydrochloride in 50% H_3_PO_4_) and incubated at room temperature for 10 min. Absorbance was then measured at 540 nm in a plate reader (Thermo, Multiskan EX).

### Flowcytometry

To measure the surface expression of co-stimulatory molecules, differentially stimulated BMDCs (1 × 10^6^/sample) were washed with phosphate buffer saline (PBS) containing 0.5% BSA and 0.1% NaN_3_ (FACS buffer) three times. After blocking, DCs were incubated with fluorochrome conjugated anti-mouse CD11c, CD80, CD83, CD86, CD40 and MHC-II antibodies for 30 min at 4 °C. Subsequently, DCs were washed, suspended in PBS and subjected to flow cytometric analysis (BD FACSCanto).

### Incubation of BMDCs with fluorescently labeled differentially charged liposomes

BMDCs were plated in 24-well flat bottom trays (Nunc) at 1 × 10^6^ cells/well. Differentially charged HPTS labeled liposomes (cationic, anionic and neutral; 100 μM) were added to triplicate wells for 18 h at 37 °C in a CO_2_ incubator. No cell toxicity was observed for the maximum incubation period. Harvested cells were washed two times with PBS and the fluorescence intensity associated with the cells were recorded at 405 and 450 nm using a fluorescence spectrophotometer (PerkinElmer, LS-55). The fluorescence associated with the same number of cells incubated with respective empty liposomes was subtracted from that associated with cells incubated with fluorescence containing liposomes for the elimination of autofluorescence. To investigate the information on the pH of the site occupied by liposomes indicating the intracellular fate of liposomes, the fluorescence emission ratio at excitation wavelengths of 450 and 405 nm was determined as described previously^59^,^60^. At these two excitation wavelengths, the fluorescence emission spectrum from HPTS shows two different peaks. The intensity of the 450 nm peak is very susceptible to pH and turns to 0 at low pH (<6.0). On the contrary, the intensity of the latter peak at 405 nm increases slightly when the pH decreases below 6.0. Thus, a higher intensity at 405 nm indicates the presence HPTS in acidic compartments and a stronger intensity at 450 nm indicates HPTS in neutral compartments. Therefore, when the fluorescence ratio of 450 nm/405 nm is higher than 1 it indicates that most of the liposomes are located in cellular compartments with pH greater than 6.0. In contrast, when most of the liposomes are located in intracellular compartments with pH lower than 6.0, the fluorescence ratio of 450 nm/405 nm is less than 1. Therefore, the intracellular fate of the liposomes can be predicted by monitoring the 450 nm/405 nm fluorescence ratio.

### Confocal immunofluorescence microscopy

Immature BMDCs were incubated with rhodamine123 labeled cationic liposomes for 20 hours. Excess liposomes were washed out, and the cells seeded in serum-free RPMI in tissue culture chamber slides. After the cells were attached to the slides (6 h incubation at 37 °C), they were fixed with 3% paraformaldehyde/PBS for 20 min and permeabilized for 15 min at room temperature by permeabilization buffer containing 10% BSA and 0.05% saponin. After that cells were incubated overnight at 4 °C temperature with antibodies against CD107a (lysosomal-associated membrane protein 1; LAMP-1), CD206 (macrophage mannose receptor), CD71 (transferrin receptor) and H2-I-A/I-E (MHC-II compartment). After two washes with permeabilization buffer, cells were incubated for 45 minutes with Texas red conjugated secondary antibodies, washed and mounted with Prolong Gold antifade reagent (Molecular probes) and examined by Andor spinning disc confocal microscope (Olympus).

### Immunization of mice and isolation of antigen-specific T cells

BALB/c mice (5–6 mice/group) were injected subcutaneously (into the lower left and right quadrant of abdomen) two times at an interval of 2 weeks with liposomal rgp63 (2.5 μg) alone or mixed with MPL-TDM (25 μg) in a total volume of 100 μl. Seven days after the second immunization spleens were removed. Single cell suspensions of the mice spleen from each group were prepared and CD4^+^ and CD8^+^ T cells were negatively separated by magnetic beading using magnetic-activated cell sorting columns (Miltenyi Biotec, Auburn, CA) according to manufacturers’ protocol.

### gp63 specific proliferation assays

Mouse BMDCs at day 7 were cultured for 18 h in the presence of empty cationic liposomes (CL; 100 μM), only rgp63 (rgp63; 2.5 μg/ml), only MPL-TDM (MPL-TDM- 100 ng/ml ), only rgp63 + MPL-TDM (rgp + MPL; rgp63- 2.5 μg/ml; MPL-TDM- 100 ng/ml), liposome encapsulated rgp63 (lrgp63; rgp63 concentration 2.5 μg/ml), or lrgp63 mixed with MPL-TDM (lrgp + MPL; lrgp- 2.5 μg/ml, MPL-TDM- 100 ng/ml). DCs cultured without stimulator were treated as control. Excess stimulators were washed out and 1 × 10^4^ DCs were co-cultured with 1 × 10^5^ CD4^+^ or CD8^+^ T cells (MACS separated CD4^+^ or CD8^+^ T cells of mice that were previously immunized subcutaneously with lrgp63 alone or mixed with MPL-TDM) in 96 well flat bottom tissue culture plates (Nunc) and T cell proliferation was assayed on day 4 by measuring the uptake (Counts Per Minute = CPM) of ^3^H-thymidine (^3^H-TdR) at a concentration of 1 μCi/well during the last 16 h of culture by scintillation counting (PerkinElmer, Tri-Carb 2800TR).

### *In vivo* T cell proliferation assays

BALB/c mice were subcutaneously immunized with a total of 2.5 μg rgp63 alone, entrapped in liposomes (lrgp) or liposomal rgp63 mixed with MPL-TDM (lrgp + MPL). In addition, mice immunized with PBS, empty cationic liposomes or with only MPL-TDM (25 μg) served as controls. After two weeks, all the animals were boosted with the same antigen preparations received initially. Five days later, draining lymph nodes (popliteal, axial and inguinal) were removed. The lymph node cell suspensions of two animals from each group were pooled separately and the CD8^+^ T cells were purified by magnetic beading (see above). 1 × 10^5^ CD8^+^ T cells were incubated with 1 × 10^4^ BMDCs that had been incubated either with liposomal rgp63 or liposomal rgp63 mixed with MPL-TDM for 18 h and T-cell proliferation was measured in a standard proliferation assay (as described above).

### Immunoblots

Stimulated mouse BMDCs were lysed in RIPA-buffer (Cell Signaling Technology, Inc.) containing protease inhibitor cocktail (Cell Signaling Technology, Inc.), incubated on ice for 30 min and debris was spun at 14,000 rpm at 4 °C. Samples (40 μg) were separated on 10% SDS-PAGE gels, transferred to PVDF membranes (Millipore) and blotted with antibodies in TBST/5% BSA to TAP1 (M-18), Tapasin (H-15), Calregulin (T-19), Gilt (T-18), Cathepsin D (C-20), Cathepsin h (N-18) (all antibodies from Santa Cruz) and β-Actin (from Sigma), and incubated with HRP-conjugated secondary antibodies (Santa Cruz). Western blots were developed using enhanced chemiluminescence (Bio-Rad, ChemiDoc MP imaging system).

### siRNA-mediated silencing of TAP-1 in BMDCs

On day 6 immature BMDCs were transfected with control siRNA (scrambled) or siRNA against TAP-1 (Santa Cruz Biotechnology, Inc.) using cationic liposomes (Supplemental method Figure S6) in reduced serum medium (Santa Cruz Biotechnology, Inc.). After 4 hours of transfection, fresh culture medium (RPMI with 10% FCS) was added and cells were stimulated simultaneously for at least 18 h. Cells were collected and screened for gene silencing by western blot analysis. Tap1 silenced and normal DCs were stimulated with liposomal rgp63 alone or mixed with MPL-TDM and co-cultured with CD8^+^ T cells (MACS separated CD8^+^ T cells of mice that were previously immunized subcutaneously with lrgp63 + MPL-TDM) and T cell proliferation was assayed by measuring the thymidine uptake (described earlier).

### Statistical analysis

Data are represented as the mean ± standard error of mean. One-way analysis of variance (ANOVA) and Tukey’s multiple comparisons post-test were used for the analysis of data using Prism-Graphpad version 5.0 (Graph pad Software, v.5.0, San Diego, CA). Student’s *t-*test was employed to assess the statistical significance of differences between a pair of data sets. *p* values of <0.05 were considered to be statistically significant.

## Additional Information

**How to cite this article**: Maji, M. *et al*. A Lipid Based Antigen Delivery System Efficiently Facilitates MHC Class-I Antigen Presentation in Dendritic Cells to Stimulate CD8^+^ T Cells. *Sci. Rep.* **6**, 27206; doi: 10.1038/srep27206 (2016).

## Supplementary Material

Supplementary Information

## Figures and Tables

**Table 1:** Particle Size, Zeta-Potential, and Antigen Entrapment Efficiency of Liposomes^a^.

**Formulations**	**Vesicle Size (nm)**	**Zeta-Potential (mV)**	**Antigen Entrapment Efficiency (%)**
Cationic Liposome	192.4 ± 10.32	60.13 ± 5.07	N/A
rgp63 in Cationic Liposome	216.7 ± 5.14	53.97 ± 10.01	58.3 ± 7.4

^a^Cationic liposomes were formulated with DSPC, cholesterol and SA (7:2:2 molar ratio) and in combination with rgp63. All data of vesicle size, zeta potential and antigen entrapment represent mean ± S.E. (n = 3). N/A- Not Applicable.

**Table 2:** Uptake of differentially charged fluorescently labeled liposomes by bone-marrow derived dendritic cells^b^.

**Liposomes**	**405 nm**	**450 nm**	**450 nm/405 nm**
HPTS-CL	400.89#	496.6	1.24
HPTS-AL	394.34	303.72	0.77
HPTS-NL	240.71	214.84	0.89

^b^BMDCs were incubated with HPTS labeled differentially charged liposomes for 18 h at 37 °C in a CO_2_ incubator. After incubation BMDCs were washed to remove excess HPTS-liposomes and the fluorescent intensity were measured at excitation wavelength of 405 nm and 450 nm. Data demonstrate 1 of 5 experiments yielding similar results. CL- Cationic liposome; AL- anionic liposome; NL- Neutral liposome.

^#^Fluorescence intensity in arbitrary unit.
