# Supplementary material for: A Lipid Based Antigen Delivery System Efficiently Facilitates MHC Class-I Antigen Presentation in Dendritic Cells to Stimulate CD8+ T Cells
Source: Sci Rep. 2016 Jun 2;6:27206. doi: 10.1038/srep27206 (PMC4890172; doi:10.1038/srep27206)
Supplement: Supplementary Information [file srep27206-s1.pdf]

Supplementary Material

A Lipid Based Antigen Delivery System Efficiently Facilitates MHC Class I Antigen Cross-Presentation in Dendritic Cells to Stimulate CD8$^+$ T-Cells

Mithun Maji,[1] Saumyabrata Mazumder,[1,2] Souparno Bhattacharya,[1,3] Somsubhra Thakur Choudhury,[1] Abdus Sabur,[1] Md. Shadab,[1] Pradyot Bhattacharya,[1] and Nahid Ali[1]

[1]Indian Institute of Chemical Biology, Infectious Diseases and Immunology Division

4, Raja S.C. Mullick Road, Jadavpur, Kolkata-700032, India.

[2]Present address: Premas Biotech Pvt Ltd, Plot No: 77, Sector 4, IMT Manesar, Gurgaon, Haryana-122050, India.

[3]Present address: University of Texas Southwestern Medical Center, Department of Radiation Oncology, Division of Molecular Radiation Biology, Dallas, Texas, USA.

"Correspondence should be addressed to N.A. (nali@iicb.res.in, nahidali28@yahoo.in)"

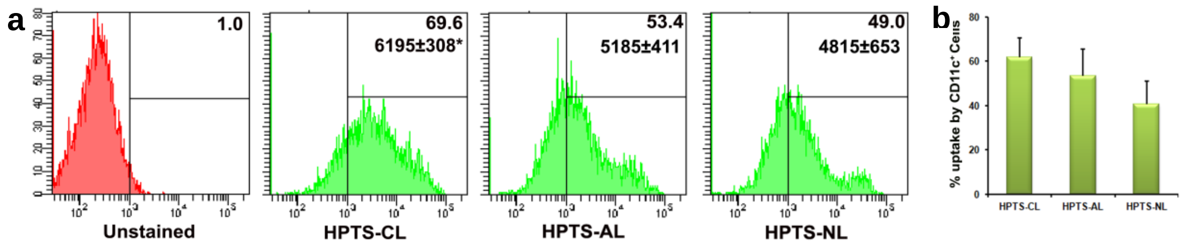

Figure S1. Differentially charged liposome uptake by bone marrow derived dendritic cells (BMDC). (a) BMDCs shows increased uptake of cationic liposomes in comparison to anionic and neutral liposomes. A representative experiment of liposome uptake by BMDCs analyzed by flow cytometry following 12 hours incubation with HPTS-labeled cationic, -anionic and -netral liposomes. Numbers inside the histogram denotes CD11c-HPTS percent positive cells (upper value) and the mean flourescence intensity ± SE (lower value), representative of three independent experiments with similar results. $*p<0.05$ analyzed by one-way ANOVA followed by Tukey's multiple comparison test, compared to all the other groups. **(b)** Percent uptake of HPTS liposomes by $CD11c^+$ BMDCs analyzed by flowcytometry (n=3). Data represent mean ± SE.

Method for Figure S1. Liposomes were labeled with HPTS as described in the manuscript. Immature BMDCs were harvested and incubated with liposomes for 12 hour in a CO_2 incubator. Harvested cells were surface stained with PE-Cy7 anti-mouse CD11c, and CD11c-HPTS positive cells were analyzed by flow cytometry.

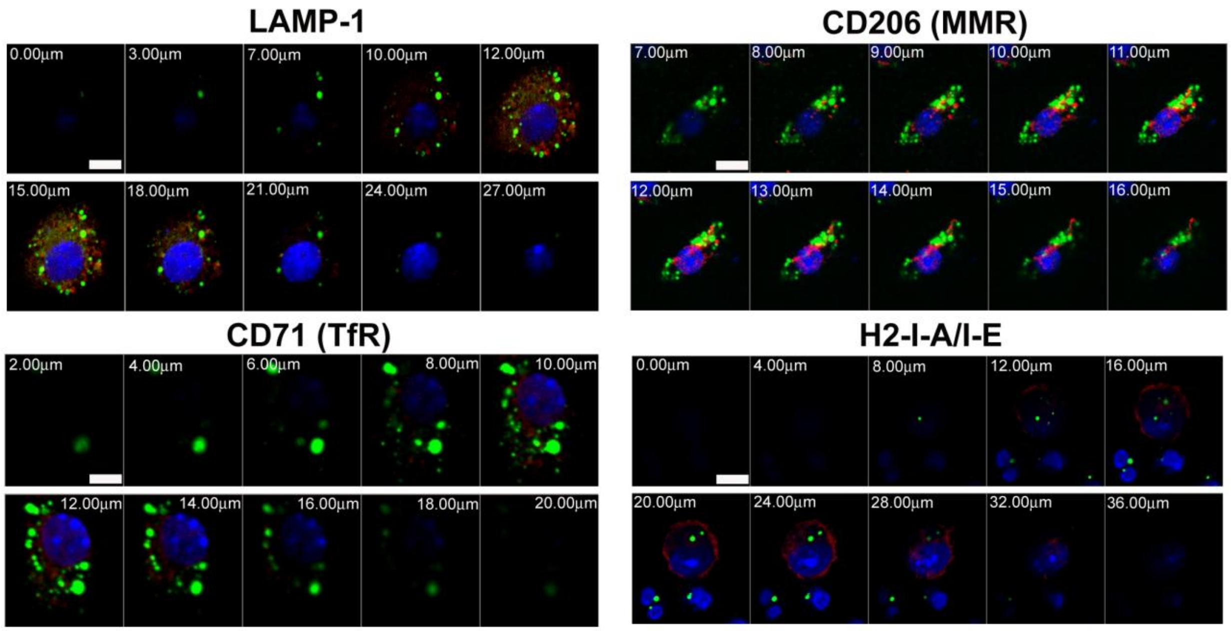

Figure S2. Intracellular trafficking of cationic liposomes in BMDCs. Mouse BMDCs were incubated with rhodamine123 labeled cationic liposomes. After incubation, unbound liposomes were washed out, and cells were mounted on slides and stained intracellularly (for details, see main manuscript "Materials and Methods") with antibodies against LAMP-1, CD206, CD71 and H2-I-A/I-E. Results are shown as rhodamine123 labeled cationic liposomes (excitation for green), antibody staining (excitation for red), and DAPI staining for nucleus of the cell (excitation for blue). Ten focal planes throughout the Z-stack of different compartments of BMDCs and location of liposomes are shown (depth of each slice is indicated in respective panels). Images were processed using Imaris 7.6.3 software. Yellow color indicates localization of liposomes with specific compartments. (Scale bar = 10 μm).

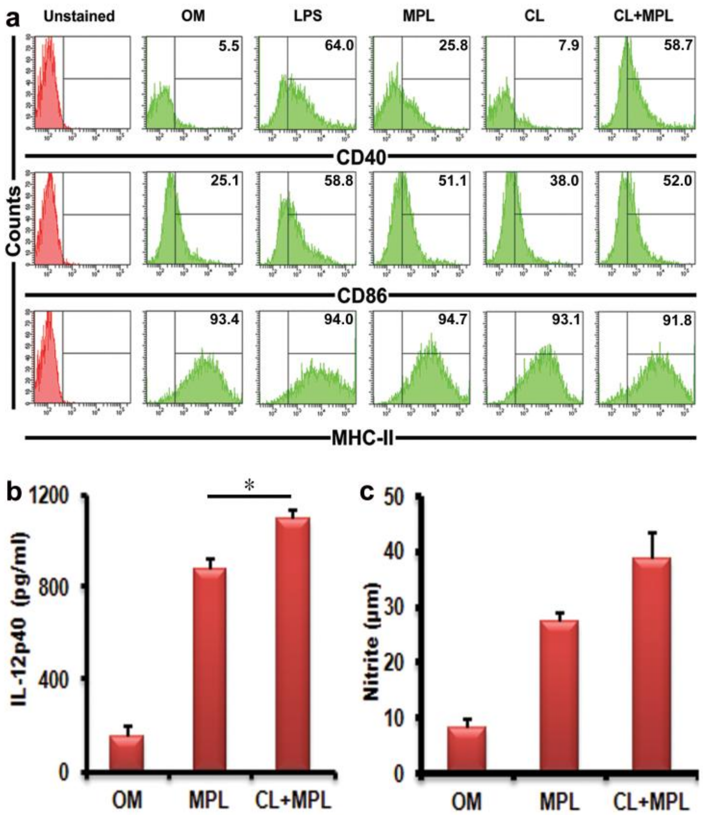

Figure S3. Adjuvanted cationic liposomes upregulated the expression of surface co-stimulatory molecules and IL-12p40 and NO secretion in BMDC. (a) Mouse BMDCs were stimulated with either TLR agonist MPL-TDM alone (MPL; 100 ng/ml), only cationic liposomes (CL; 100 μM), cationic liposomes mixed with MPL-TDM (CL+MPL; CL- 100 μM, MPL- 100 ng/ml) or with LPS (served as positive control). Subsequently, treated DCs were stained with fluorochrome conjugated anti-mouse antibodies and analyzed by flow cytometry. The numbers inside the histogram is the percent positive populations of the indicated surface markers on CD11c+ BMDCs upon various treatments. Rows display different surface markers, and columns display different compounds used for stimulation. Results are representative of one of the three experiments performed. **(b, c)** Mouse BMDCs were stimulated with either TLR agonist MPL-TDM alone (MPL; 100 ng/ml) or mixed with cationic liposomes (CL; 100 μM). Unstimulated

BMDCs served as control. Culture supernatants were removed at 24 h for IL-12*p*40 quantification and at 72 h for measurement of nitric oxide (NO) production using Griess reagent. Data represent triplicate mean ± SE (n=3), representative of three independent experiments with similar results. $*p<0.05$ analyzed by one-way ANOVA followed by Tukey's multiple comparison test.

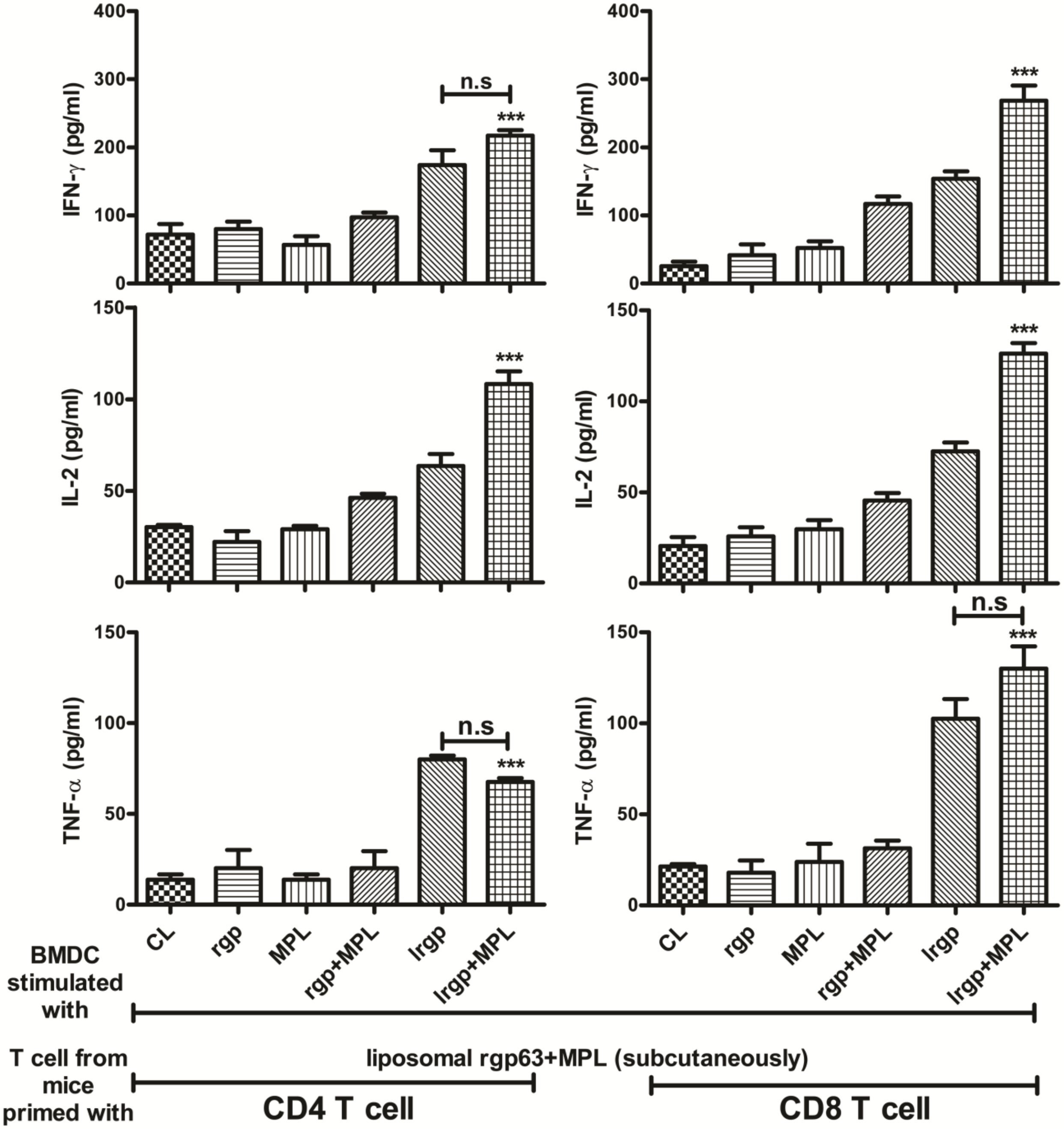

Figure S4. Adjuvanted liposomal protein induced cytokine production from T cells. BMDCs were stimulated with 2.5 µg of rgp63 free, entrapped in liposomes or liposomal rgp63 mixed with MPL-TDM. BMDCs stimulated with empty cationic liposomes (CL), only MPL-TDM, only rgp63 or rgp63+MPL-TDM served as controls. After 20 h $1x10^4$ BMDCs were co-cultured with $1x10^5$ $CD8^+$ T cells isolated from mouse immunized with lrgp+MPL-TDM. After 4 days of culture, IFN-γ, IL-2 and TNF-α were measured in the collected culture supernatants.

Data represent mean ± S.E. (n=3); ***p<0.001 analyzed by one-way ANOVA followed by Tukey's multiple comparison tests, compared to all the other groups. n.s- not significant.

Method for Figure S4. Mouse BMDCs at day 7 were cultured for 18 h in the presence of empty cationic liposome (CL; 100 µM/ml), rgp63 (rgp63; 2.5 µg/ml), liposome encapsulated rgp63 (lrgp63; rgp63 concentration 2.5 µg/ml) or lrgp63 mixed with MPL-TDM (lrgp+MPL). DC cultured without stimulator, with only MPL-TDM, and rgp63+MPL-TDM were treated as controls. Excess stimulators were washed out and 1 x 10^4 DCs were co-cultured with 1 x 10^5 $CD4^+$ and $CD8^+$ T cells (MACS separated $CD4^+$ and $CD8^+$ T cells from mice that were previously immunized subcutaneously with lrgp63 mixed with MPL-TDM) in 96 well flat bottom tissue culture plates (Nunc). Culture supernatants were collected after 4 days of culture for cytokines measurement through ELISA, as detailed in the instructions provided with cytokine ELISA kits (eBiosciences).

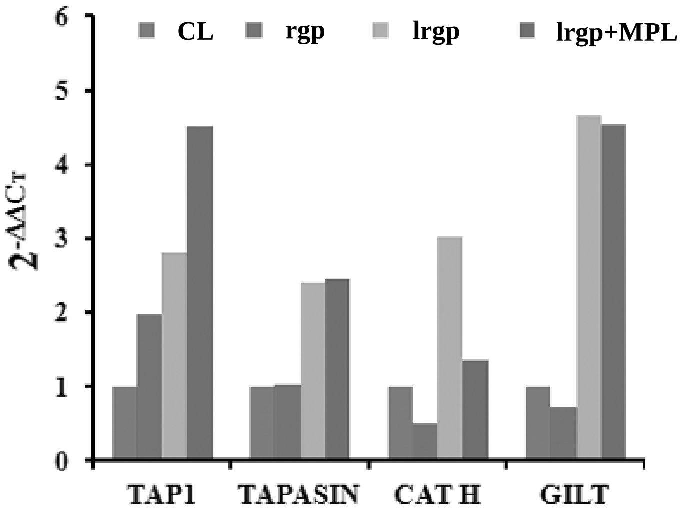

Figure S5. Expression of genes associated with MHC class-I and MHC class-II pathways of antigen processing machinery by differentially stimulated BMDCs. BMDCs were stimulated with only gp63, liposomal gp63 and liposomal gp63+MPL-TDM. DCs stimulated with only liposome (CL) served as controls. RNA isolated from the stimulated DCs was subjected to real-time analysis, and GAPDH mRNA levels were used for normalization. Data represent triplicative mean of one individual experiment of the three experiments yielding similar results.

Method for Figure S5. Total RNA was isolated from stimulated BMDCs ($1x10^6$ cells) with Trizol Reagent and purified on Qiagen RNeasy Mini Kit column. DNase digestion was carried out during RNA extraction (RNase-free DNase Set, Qiagen). Spectrophotometrically evaluated RNA was reverse transcribed with random primers (cDNA Reverse Transcription Kit, Applied Biosystems) and qRT-PCR was performed on cDNA from independent samples, using primer sets specific for selected genes and the GAPDH housekeeping gene. qRT-PCR was carried out on a 7500 machine (Applied Biosystems) with Power SYBR Green PCR Master Mix (Applied Biosystems). Assays were performed in duplicate. Relative mRNA levels were calculated by the $2^{-\Delta\Delta Ct}$ method ($\Delta Ct = Ct_{target} - Ct_{GAPDH}$, $\Delta\Delta Ct = \Delta Ct_{stimulated} - \Delta Ct_{untreated}$), using GAPDH as housekeeping gene.

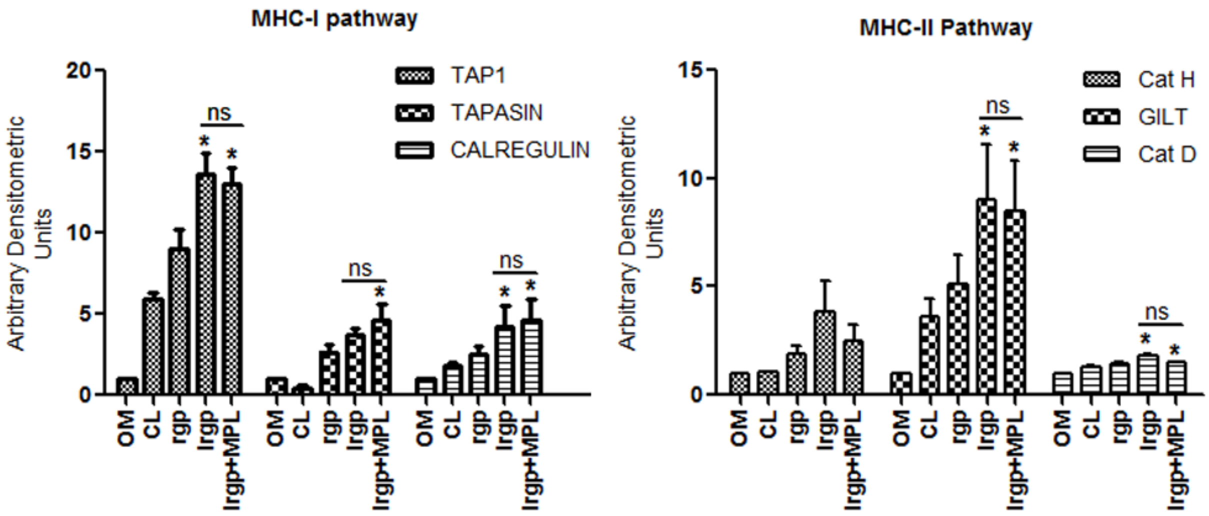

Figure S6. Expression of molecules associated with MHC class-I and MHC class-II pathways of antigen processing machinery by differentially stimulated BMDCs. BMDCs were stimulated with only gp63, liposomal gp63 and liposomal gp63+MPL. Unstimulated DCs (OM) and DC stimulated with only liposome (CL) served as controls. β-actin was used as loading control. Band intensities were calculated using ImageJ software, normalized to the housekeeping gene β-actin, and assigned values relative to the corresponding only media (OM) control, which was given a value of 1 for ease of comparison. Data represent Arbitrary Densitometric Units mean ± SE (n=3); $*p<0.05$ analyzed by one-way ANOVA followed by Tukey’s multiple comparison tests, compared to all the other groups. ns- Not significant.

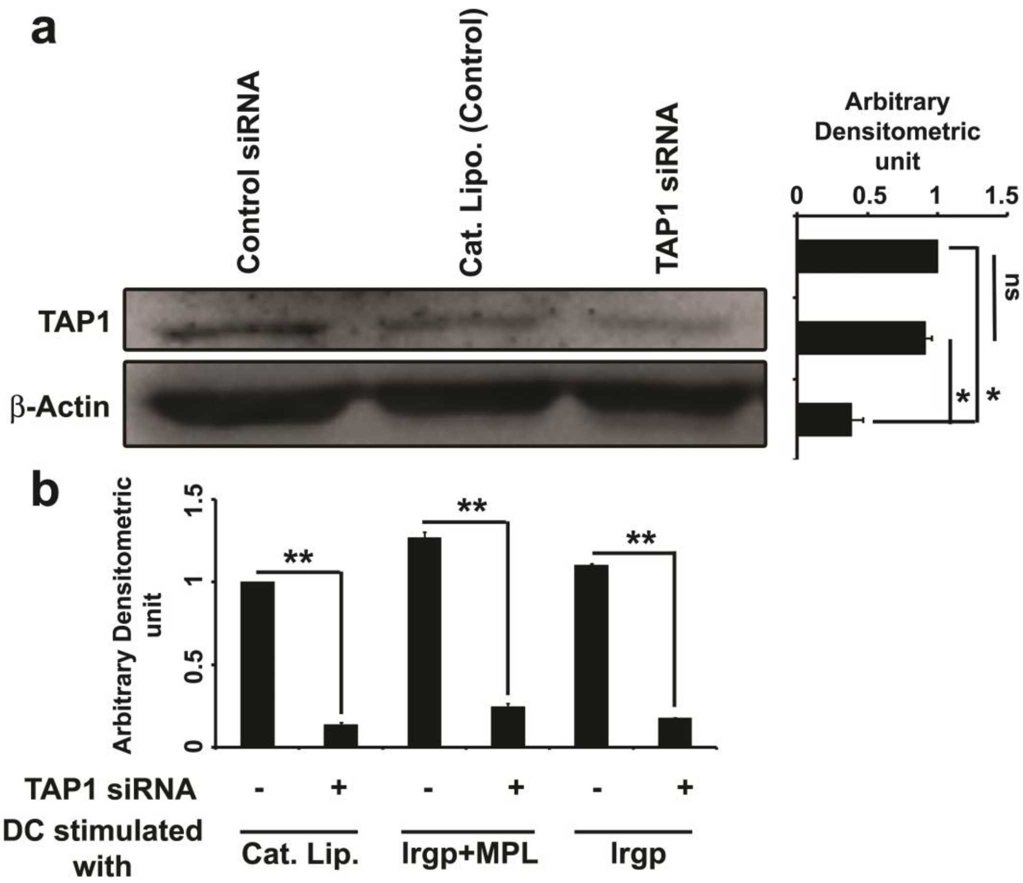

Figure S7. knockdown of TAP1 gene by siRNA in DCs. BMDCs were transfected with control siRNA (scrambled) or siRNA against TAP-1 in reduced serum medium. After 4 hours of transfection, fresh culture medium was added and cells were allowed to grow normally for at least 14 h. DC stimulated with only cationic liposomes served as experimental control. β-actin was used as loading control. **(a)** Expression of TAP1was evaluated by western blot analysis. The ratios of intensities of particular bands/loading controls are indicated as bar graphs adjacent to the figure. $*p<0.05$ analyzed by one-way ANOVA followed by Tukey's multiple comparison tests, compared to all the other groups. **(b)** Expression of TAP1 abrogation in siRNA treated BMDCs were evaluated by Western blot analysis (Main Manuscript Figure 6A)**.** Band intensities were calculated using ImageJ software (version 1.42), normalized to the housekeeping gene β-actin, and assigned values relative to the only cationic liposome (Experimental control), which was given a value of 1 for ease of comparison. $**p<0.01$ analyzed by Students *t*-test.

Method for Figure S7. siRNA encapsulation in liposomes and transfection. Briefly, lipid mixtures (DSPC, cholesterol and SA at a molar ratio of 7:2:2) were dissolved in methanol-

chloroform solution and the mixture was evaporated to dryness in a round bottom glass flask to make a thin lipid film. Empty and siRNA entrapped liposomes were prepared by dispersion of lipid film in either 1ml of PBS alone or containing siRNA (Scramble or TAP1; 1µM/ml in PBS). The mixture was then vortexed and sonicated in an ultrasonicator (Misonix; New York, USA) for 30 s, followed by incubation at 4 ºC for 2 h. 100 µM of siRNA encapsulated liposomes were added with 30 pmol of siRNA and kept at room temperature in serum free medium for 20 min before transfection of the cells in culture medium (absence of serum/antibiotic).

Table S1. Characterization of conventional liposomes[c]

Formulations	Vesicle Size (nm)	Zeta-Potential (mV)
**Anionic Liposomes**	405.5 ± 51.58	-72.07 ± 1.69
**Neutral Liposomes**	312.8 ± 24.08	-4.379 ± 2.59

[c]Neutral and anionic liposomes were formulated with DSPC and cholesterol (7:2 molar ratio) or DSPC, cholesterol and PA (7:2:2 molar ratio), respectively. All data of vesicle size, zeta potential represent mean ± S.E. (n=3).

Table S2. Primers used for real time PCR.

Target	Forward Primer	Reverse primer
TAP 1	5'-GCGCTGGATTACTGTACTGGAA-3'	5'-GGCTGCGGGCAATGC-3'
TAPASIN	5'-ACACCAGCACCCGTTGTGT-3'	5'-GAAGAAGTGGGATGCAAGACAGA-3'
Cathepsin H	5'-AACAATCATGGCTGCAAAGGA-3'	5'-TCTTCCATGATGCCCTTGTTG-3'
GILT	5'-TCCCTTTCGCAAGCATCCT-3'	5'-CCCAAGAGGCACACATCGT-3'
GAPDH	5'-CATGGCCTTCCGTGTTCCTA-3'	5'-CCTGCTTCACCACCTTCTTGAT-3'

Post imaging Analysis. All western blot images were processed with Adobe Photoshop 7 for linear adjustments and cropping. All images were captured on Olympus microscope and cropping was done using Adobe Photoshop 7.
